# The Tenure of Hospital Staff Appointments

**Published:** 1913-07-05

**Authors:** 


### THE TENURE OF HOSPITAL STAFF APPOINTMENTS.

### Staff Elections in the United States.

The problems raised by the case of Eddowes and Others *v.* St. John's Hospital are illustrated by the way in which staff elections are managed in the States.

In America different systems have been experimented with. One which appears to have answered very well in some of the new hospitals and schools, which have been born full-grown, as it were, in the United States through the munificence of individual founders, is to have one medical man as supreme head of each separate department—such as surgery, medicine, gynæcology, and the rest. Working under this chief are two or three or four "associates," men who correspond to members of the visiting staff with us. Under each associate, furthermore, work one or two assistants, corresponding to the out-patient staff as we know it.

Thus, instead of a staff each member of which is a law unto himself, and absolute autocrat of his own wards, this American system supplies a chain of command like that of an army, in which each worker is responsible to some superior, except the heads of departments, who are responsible to the governing body. In this way there is no question but that research and teaching flourish amazingly; though we are not saying that the system is suitable for adoption on this side of the Atlantic, where conditions are so totally different and institutions grow slowly to maturity, instead of springing up full-sized and fully endowed in a moment.

### A Chicago Experiment.

In one of the Chicago hospitals a very extraordinary experiment in staff election, or re-election, has been tried, and, according to report, with great success. Dissatisfied with certain members of their staff, the Governors of the Cook County Hospital planned a clean sweep of the staff, and an interesting competitive examination for the vacant places. For this examination any qualified man could sit, and was set to answer searching examination papers in whatever specialty he professed. In order to equalise matters between young men of no practical experience, fresh from their schools, and older men with experience and technique already acquired, the highest possible marks for the examination papers were 60 per cent. of the total marks to be awarded. The other 40 per cent. of marks were allotted for what was called "experience." In calculating a candidate's marks for this asset, regard was paid to the number of years already served on the staff, the original work published (if any), the amount of time devoted to teaching students, and several other similar factors.

Thus a senior man with a good reputation had only to do moderately well on the examination papers to retain his post; and yet it was not impossible for a really brilliant junior to aspire to some post held by an incompetent senior whose continuance in office blocked the way to promotion. It may be suspected that the system's greatest success as a rule would be in keeping the seniors up to the mark rather than in ousting them in favour of juniors; but, at any rate, the Governors of the hospital seem to have been highly satisfied with the result in this particular case.

Needless to say, this extraordinary process of reviving the examination bugbear of their younger days did not appeal at all to the imagination of the medical profession, which denounced the proposal in round terms. In Great Britain, we imagine, such a system would be impossible to establish, though the mere contemplation of such a thing suggests some amusing reflections. What a spectacle, for instance, would be provided by Sir Victor Horsley, let us say, being examined in brain surgery, or Dr. Bulloch in bacteriology, or Sir John Bland Sutton in gynæcology, to mention only three personalities of the day in their respective specialties! And who would be temerarious enough to undertake the examination of such experts? *Quis custodiet ipsos custodes?*

Seriously, the chance of our following the Chicago example may confidently be regarded as absolutely *nil.* But to say that governing boards of voluntary hospitals cannot dispense with the services of their medical officers, save for serious misconduct, is a proposition which strikes at the root of the whole basis of hospital management.

